# Stable, simultaneous and proportional 4-DoF prosthetic hand control via synergy-inspired linear interpolation: a case series

**DOI:** 10.1186/s12984-021-00833-3

**Published:** 2021-03-18

**Authors:** Platon Lukyanenko, Hendrik Adriaan Dewald, Joris Lambrecht, Robert F. Kirsch, Dustin J. Tyler, Matthew R. Williams

**Affiliations:** 1 Department of Biomedical Engineering, Case Western Reserve University, 10900 Euclid Avenue, Cleveland, OH 44106-1712 USA; 2 Cleveland FES Center, Louis Stokes Cleveland Veterans Affairs Medical Center, 10701 East Boulevard, B-E210, Cleveland, OH 44106-1702 USA; 3 APT Center, Louis Stokes Cleveland Veterans Affairs Medical Center, 10701 East Blvd., Mail Stop 151 W/APT, Cleveland, OH 44106-1702 USA

**Keywords:** Electromyography, Prosthetic control, Virtual reality, Interpolation

## Abstract

**Background:**

Current commercial prosthetic hand controllers limit patients’ ability to fully engage high Degree-of-Freedom (DoF) prosthetic hands. Available feedforward controllers rely on large training data sets for controller setup and a need for recalibration upon prosthesis donning. Recently, an intuitive, proportional, simultaneous, regression-based 3-DoF controller remained stable for several months without retraining by combining chronically implanted electromyography (ciEMG) electrodes with a K-Nearest-Neighbor (KNN) mapping technique. The training dataset requirements for simultaneous KNN controllers increase exponentially with DoF, limiting the realistic development of KNN controllers in more than three DoF. We hypothesize that a controller combining linear interpolation, the muscle synergy framework, and a sufficient number of ciEMG channels (at least two per DoF), can allow stable, high-DoF control.

**Methods:**

Two trans-radial amputee subjects, S6 and S8, were implanted with percutaneously interfaced bipolar intramuscular electrodes. At the time of the study, S6 and S8 had 6 and 8 bipolar EMG electrodes, respectively. A Virtual Reality (VR) system guided users through single and paired training movements in one 3-DoF and four different 4-DoF cases. A linear model of user activity was built by partitioning EMG feature space into regions bounded by vectors of steady state movement EMG patterns. The controller evaluated online EMG signals by linearly interpolating the movement class labels for surrounding trained EMG movements. This yields a simultaneous, continuous, intuitive, and proportional controller. Controllers were evaluated in 3-DoF and 4-DoF through a target-matching task in which subjects controlled a virtual hand to match 80 targets spanning the available movement space. Match Percentage, Time-To-Target, and Path Efficiency were evaluated over a 10-month period based on subject availability.

**Results and conclusions:**

In 3-DoF, S6 and S8 matched most targets and demonstrated stable control after 8 and 10 months, respectively. In 4-DoF, both subjects initially found two of four 4-DoF controllers usable, matching most targets. S8 4-DoF controllers were stable, and showed improving trends over 7–9 months without retraining or at-home practice. S6 4-DoF controllers were unstable after 7 months without retraining. These results indicate that the performance of the controller proposed in this study may remain stable, or even improve, provided initial viability and a sufficient number of EMG channels. Overall, this study demonstrates a controller capable of stable, simultaneous, proportional, intuitive, and continuous control in 3-DoF for up to ten months and in 4-DoF for up to nine months without retraining or at-home use with minimal training times.

**Supplementary Information:**

The online version contains supplementary material available at 10.1186/s12984-021-00833-3.

## Background

At present, an outstanding challenge in biomedical engineering is the restoration of hand function to upper limb amputees. Current commercially available myoelectric hand prostheses are typically limited to one or two Degrees of Freedom (DoF) and experience a 10–35% abandonment rate [1]. The man–machine interface, limited in both feed-forward control and closed-loop control, is a major contributor to abandonment [2, 3]. Advanced prosthetic systems, such as the 10-DoF DEKA/LUKE Arm [4] and the 16-DoF Modular Prosthetic Limb [5], have expanded prosthetic hand capabilities, but place even higher demands on prosthetic hand controllers.

Recent work has demonstrated the potential of chronically implanted man–machine interfaces. Chronically implanted nerve electrodes have shown stable sensory feedback [6–9], and chronically implanted Electromyographic (ciEMG) electrodes have demonstrated stable, low-crosstalk EMG recording capabilities [10]. Using ciEMG to improve feed-forward EMG controllers could allow users to gain more functional benefit from both advanced and commercially available prosthetic devices.

### Feed-forward EMG controllers

Feed-forward EMG controllers interpret user EMG to drive a prosthetic hand, and typically involve three main steps. First, raw EMG is recorded, filtered, and windowed into sections of 100–200 ms. Then, features such as the mean-absolute-value or number of zero crossings are extracted from the windowed EMG. Lastly, features are mapped to hand velocities. Prior work suggests that feed-forward EMG controllers should meet several criteria to provide natural hand control (Table 1) [11–19].

**Table 1:** Natural hand control criteria [11–19]

Category	Criteria (a controller providing natural hand control should…)
Runtime	be responsive; processing time < 100 ms
Theoretical Capability	have many Degrees of Freedom (DoF)
	be Proportional; allow variable hand speed
	allow Simultaneous DoF activation
	be Continuous; not limit simultaneous DoF activations to fixed ratios
Accuracy	have a low error rate
Practicality	provide Intuitive control
	have a low daily set-up time
	require infrequent training
Impact	be functionally beneficial
	be tolerant to daily use
	address a large commercial audience

The terms proportional, simultaneous, and intuitive commonly appear in literature. Continuous controllers typically appear in literature as regressions and contrast with classifiers, which only allow movement in fixed, pre-defined DoF ratios. Studies often increase the number of movement classes rather than the number of continuously-controlled DoF

### Recent studies demonstrate intuitive, simultaneous, proportional controllers

Intuitive prosthetic hand control has been widely adopted since its introduction by (Hudgins 1993) [20]. Intuitive controllers are generated by recording a sample of user EMG, a training data set, and tailoring controller behavior to the user based on this sample. Studies have demonstrated that intuitive controllers, generated by machine learning methods, are capable of simultaneity, allowing combined movements, and proportionality, allowing hand speed to change with user effort, as 3-DoF classifiers [9, 18, 21]^.^ Continuous controllers have also been demonstrated through 2-DoF [12, 22]_,_ and very recently, 3-DoF [10] regressions. In this work we propose a novel simultaneous, continuous, intuitive, and proportional controller for 4 + -DoF.

### Higher DoF control is limited by training data collection

One limit on simultaneous, intuitive, proportional 3 + -DoF prosthetic hand control is training data acquisition. Gathering and labelling sufficient volumes of data is particularly problematic for simultaneous high DoF controllers: providing a single complete training set for a simultaneous 4-DoF controller, for example, would require sampling 80 non-rest movement combinations and, at 30 s per movement, correspond to a 40-min exercise. Training periods of this length are impractical for commercial systems and unduly burden users. As a result, training data is often limited to individual movements or limited simultaneous combinations (Young 2013) [14]. Training data collection time can be reduced either by lowering the controller’s reliance on data volume or by reducing the frequency of controller re-training [21, 23–25].

### Synergy Theory can reduce training data volume

Synergy Theory is a framework for describing muscle activity which states that muscles are driven in synergies, sub-movements in which individual muscles are contracted in fixed ratios commanded by a common neural signal [26]. The formulation of Synergy Theory used in this study describes synergies as time-invariant and assumes that EMG signals are at steady state and that only the mean-absolute-value (mABS) feature is used. Under these assumptions, the EMG signal for a movement is a linear combination of EMG from underlying sub-movements. Synergy Theory can be used to allow control over complex movements from small training sets [23]_,_ which is typically done by identifying synergies and using them to control individual DoF [23, 27, 28]. This work instead engages Synergy Theory to motivate the choice of controller algorithm and training data set.

Users have direct control over synergy magnitudes, and synergy magnitudes relate linearly to force, a proxy measurement for user effort [29]. This implies that user movements have unique steady-state EMG patterns. It is therefore unnecessary to sample movements at several levels of effort if the controller used is a mapping homogeneous in degree one (e.g. a linear map). Also, if the number of synergies used equals the number of EMG channels, the mapping from EMG feature space to synergy space is an orthogonal change of basis. Under these conditions, mappings such as linear regression and linear interpolation implicitly operate in synergy space. Such mappings would also implicitly implement proportionality using synergy magnitudes, which has been hypothesized to reduce user effort compared to traditional mappings [29].

### Linear interpolation follows from linear regression studies

Linear regression for prosthesis control has been examined in past work [15, 22, 30]. Notably, Nowak and Castellini [31] found that linear regression performance improves if un-trained multi-DoF movements are approximated by linear combinations of trained single-DoF movements. Additionally, Nowak and Castellini found that non-linear regression methods are more accurate than linear regression (although importantly proportionality was not evaluated).

In this work, we have developed a controller that uses linear interpolation to map EMG mABS features to user intent. Linear interpolation implicitly combines the EMG of trained movements to predict the EMG of un-trained movements. Linear interpolation is also piecewise-linear, presenting a middle ground between non-linear and linear approaches. Interpolation estimates outputs from a set of input–output pairs. Input space is partitioned into regions bounded by the sample inputs, outputs are predicted by linearly interpolating partition vertices. In the context of EMG, this can be accomplished by loosely viewing all steady-state EMG for a trained user movement as a point in EMG feature space. This lumps training repetitions together and ties controller performance to the repeatability, rather than volume, of training data. The controller’s goal is, from a collection of such irregularly spaced points, to determine a user’s movement given a new signal in EMG feature space. These methods have been extensively developed in other fields [32, 33]. Linear interpolation is best compared to regression controllers, as the output of interpolation is continuous. An important distinction between linear interpolation and linear regression is that interpolation fits user data exactly rather than in a least-square sense: if a user recreates a training movement exactly, interpolation will always provide the correct movement.

### Stability of chronically implanted EMG

Chronically implanted EMG (ciEMG) has been shown to reduce the need for frequent controller re-training, as recently demonstrated 3-DoF simultaneous, intuitive, regressive, proportional controller employing a K-nearest-neighbor mapping that retained performance in a posture-matching task over several months [10]. Separately, ciEMG has also been shown to improve user performance with standard tests and reduce fatigue [2].

Additionally, combining the synergy framework and ciEMG can potentially reduce user effort associated with implementing proportional control. Proportionality is often implemented by scaling prosthesis speed by the average magnitude across all EMG recordings (e.g. Simon 2011 [19]). This is less effective with ciEMG, where a user’s movement might only manifest on a single EMG channel. Instead, synergy magnitudes can be used to implement proportionality [30]. This work engages ciEMG recordings from two subjects.

### Hypothesis

In this work we propose a novel, intuitive, simultaneous, proportional and continuous controller for prosthetic hands. The controller uses ciEMG recordings to model user activity through linear interpolation as inspired by the synergy framework. The controller is evaluated in an online Virtual Reality (VR) posture-matching task.

In particular, we hypothesize that, provided a sufficient number (at least two per DoF, see Methods) of relevant ciEMG channels, (1) subjects will be able to use such a controller to match most targets in 3-DoF and 4-DoF and (2) that controllers will remain stable, showing no decline in performance for more than 6 months without retraining. Additionally, we explore controller performance when trained on a single ‘best’ training repetition for each trained movement, with the hypothesis that performance will remain unchanged compared to a default training set.

## Methods

Two subjects with unilateral, transradial limb loss, S6 and S8, participated in the study. Both were previously implanted with 8 pairs of intramuscular myoelectric signal (IM-MES [34]) recording electrodes, accessible through percutaneous leads. After recording training data, a controller incorporating a synergy framework was developed and evaluated through a posture-matching task. Controllers were evaluated in lab over 8–10 months and varied by subject availability. Between lab sessions, subjects used a single-DoF prosthesis controlled by two surface myoelectric sites as provided by their prosthetist. All research was conducted as an IDE trial granted by the Food and Drug Administration and under approval and oversight by the Louis Stokes Cleveland Veterans Affairs Medical Center Institutional Review Board, and the Department of Navy Human Research Protection Program.

### Data and training

Seven bipolar IM-MES electrodes were implanted in the pronator, FCR, FDS, FCU, supinator, ED, and ECU muscles of both subjects. An eighth electrode was placed in the ECRL for S6 and the ECRB for S8. (Dewald [10]) describes surgical details and stable 3-DoF controller performance over several months without retraining in S6. After fitting new sockets on S6, electrodes in the pronator and ECRL exhibited considerable noise 11 and 17 months post-implant, respectively, and were not used in this study.

**Fig. 1:**
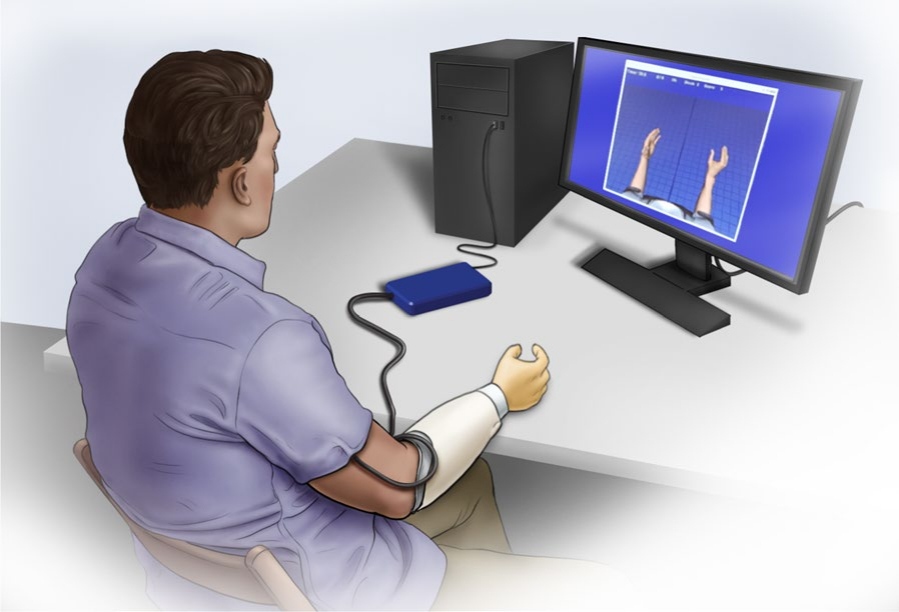
Training and testing setup. The subject was seated in front of a VR representation of a prosthetic hand. The Ripple Grapevine system collected ciEMG at 2 kHz with a 15–350 Hz band-pass filter. The only EMG feature collected was the mean absolute value over a 200 ms window updated every 50 ms

A computer visualization (Fig. 1) previously used in similar studies [35] guided the acquisition of training data. The visualization displays two hands whose joints can be controlled in real time through a Matlab/Simulink interface. The controllable joints mimic the capabilities of the LUKE prosthetic hand. During training, both hands present target postures guiding the user through a set of movements. During online evaluation, one hand presents a target posture, while the other is under the user’s control.

Five training sets were collected per subject: one 3-DoF set and four 4-DoF sets. The movements collected for each training set were determined by which DoF were included. All training sets included three DoF from (Dewald 2019): wrist flexion/extension, wrist pronation/supination, and D2 (index) flexion/extension. The 4-DoF sets also included either thumb flexion/extension, thumb ab/adduction, synchronous D3-5 flexion/extension, or wrist radial/ulnar deviation as the 4th DoF. These DoF reflect the capabilities of the LUKE prosthetic hand (Table 2). Visually, radial-ulnar deviation was mapped to a thumb movement as the LUKE is not capable of controlling this motion independently. Additionally, when synchronous D3-5 flexion/extension was not evaluated, user D2 (index) flexion/extension was made to control fingers D2-5. While this does not allow meaningful grasps in 3-DoF, it limits user confusion during controller evaluation. In practice, D2 flexion/extension can be mapped to a grasp.

**Table 2:** Usability of trained controllers

		User training sets				
		3-DoF	4-DoF	4-DoF	4-DoF	4-DoF
DoF Trained	Wrist Pro/Supinate	√	√	√	√	√
	Wrist Flex Extend	√	√	√	√	√
	D2 Flex Extend	√	√	√	√	√
	D3-5 Flex Extend		√			
	Thumb Ab/Adduct			√		
	Thumb Flex/Extend				√	
	Radial-Ulnar Deviate					√
Usable	S6 Controllers	3D			TH	RU
	S8 Controllers	3D		TH		RU

Subjects could deem a controller unusable during controller tuning and testing. Each subject deemed the 3-DoF (3D) and two of four 4-DoF (the radial-ulnar movement RU, and a thumb movement, TH) controllers usable

To generate training data, subjects attempted to move their phantom limb as prompted by a visualization at a self-selected medium level of exertion while mirroring the motion with their intact limb. Every N-DoF training set consisted of individual movements ( 2(N) movement classes: two directions (‘ + ’, ‘−’) per DoF) and all simultaneous pairs of movements (4(N!/(2!(N-2)!)) movement classes: four directions (‘ + / + ’, ‘+/-’, ‘−/ + ’, ‘−/−’) for each DoF pair). Training was done in batches of 5–10 movement prompts, grouped by movement similarity and complexity. Each batch was repeated five times with random presentation order to collect five repetitions of each movement. To mitigate fatigue, subjects could choose to take short breaks following each movement batch. Subjects determined break length, and typically chose to proceed without one. Each movement prompt had three periods: a two-second no-movement visualization period to recognize the target posture, a two-second muscle contraction period to move the phantom, and then a one-second break period. Screen color changes indicated period transitions. To ensure that subjects followed the intended movement, a researcher mimicked this exercise while sitting next to the subject and watching his intact limb.

Total training time for all five training sets was approximately 135 min including breaks, producing one 18-movement 3-DoF set and four 32-movement 4-DoF sets (Table 2). Training data was recorded 23 months post-implant for S6, and 12 (3-DoF) and 17 (4-DoF) months post-implant for S8. Both subjects had substantial experience with training data collection and wore their regular prosthesis during both data collection and controller evaluation. The only EMG feature collected was the mean-absolute-value feature from a 200 ms window of EMG sampled at 2 kHz, updated every 50 ms.The mean-absolute-value EMG feature on each channel was normalized to a maximum of ‘1′ per training data set [36]. A visualization of the ciEMG patterns for the S8 3-DoF training set (18 movements, 5 repetitions of each) is provided as Additional file 1.

### Channel sufficiency

A previous study, (Muceli 2010 [37]), found that 2-DoF multi-DoF movements can be accurately reconstructed using four, but not three, synergies extracted from single-DoF movements. The same could also be achieved with only three synergies extracted from multi-DoF movements, but depended strongly on the choice of sampled movements. As we cannot guarantee that the movements sampled in this study meet the second criteria, Channel Sufficiency is defined as twice the number of DoF.

### Controller

The controller used in this study uses linear interpolation to map user ciEMG to intended prosthesis movement. Prior to controller generation, the five repetitions of each movement are limited to steady state activity, here defined as a half-second interval beginning one second after the ‘go’ instruction. Additionally, two repetitions with EMG patterns most distant from the movement mean are removed to account for errors during user training.

#### Partitioning space (Fig. 2a, b)

**Fig. 2:**
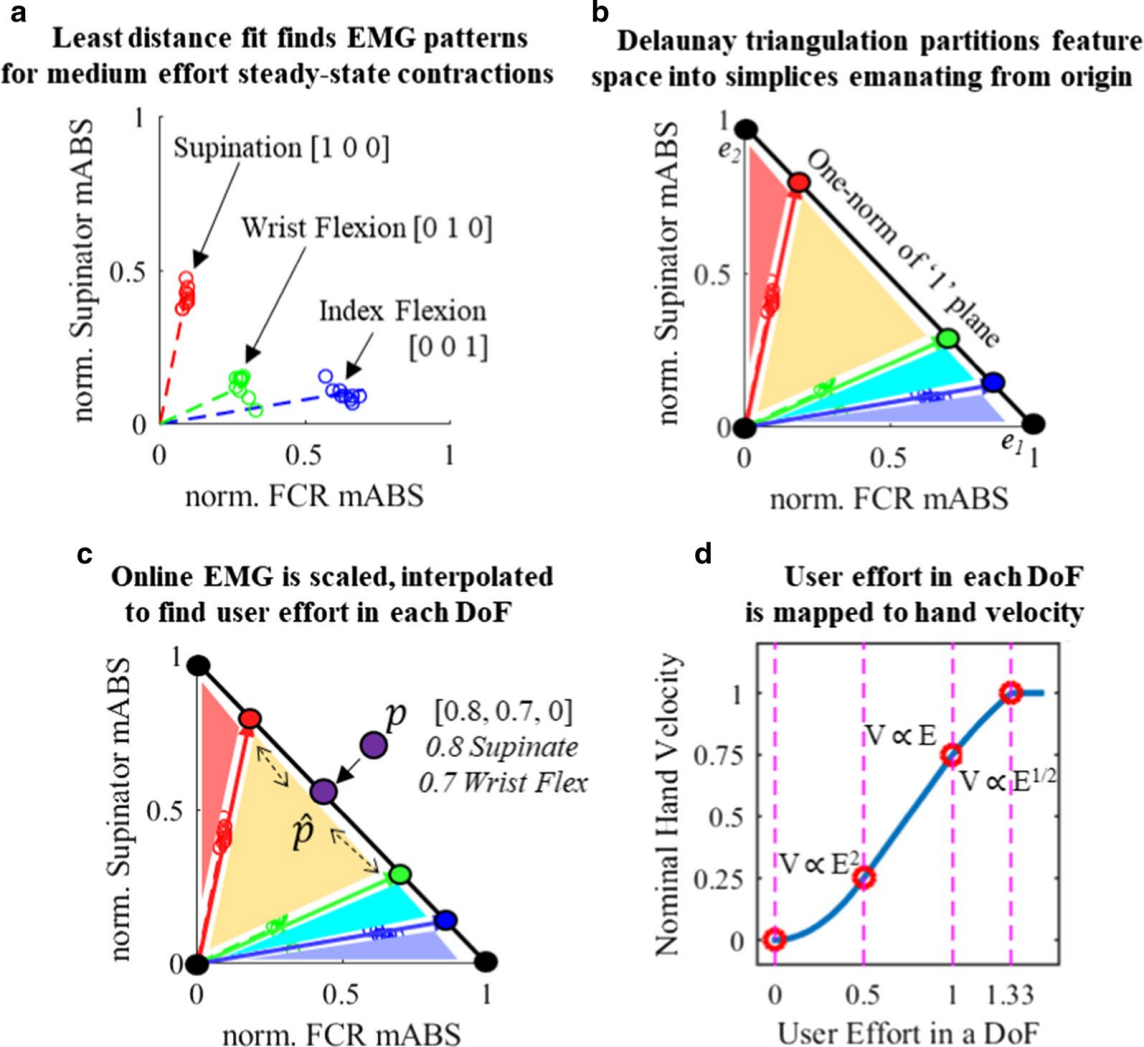
Graphical description of controller. a Per trained movement, a representative steady-state point in feature space is found and assigned a movement label b Representative points are normalized to have unit L^1^ norm, and Delaunay Triangulation partitions feature space into regions emanating from the origin c The movement label for online EMG is determined by linear interpolation, providing an estimated level of user intent in every DoF (D) A physiologically inspired relation maps estimated levels of user intent to nominal hand velocity. Subject preferences then set gains and thresholds

*Fit steady state.* Per trained movement, a vector is drawn through the cluster of steady state EMG points via a least-distance fit (uncentered 1st principal component). A point, ${\varvec{s}}$, is then found by projecting the movement’s steady-state EMG points onto this vector, then averaging their positions. ${\varvec{s}}$ is representative of its movement’s steady state EMG pattern at a medium level of exertion. ${\varvec{s}}\in {R_{\ge 0}^{n}}\text{;}\, n \text{ is the number of EMG channels; } {\varvec{S}}\text{ is the set of all } {\varvec{s}} \text{ in some fixed order}$.

*Assign movement labels.* For each ${\varvec{s}}$ a corresponding movement label ${\varvec{c}}$ is made. ${\varvec{c}}$ is an array encoding the user’s movement that generated ${\varvec{s}}$: the values in ${\varvec{c}}$ represent the direction and level of effort in each DoF. (Ex: [1,0,0] is the label for supination in 3-DoF; [− 1,0.3,0,0] is the label for a simultaneous pronation (negative supination) and slight wrist flexion in 4-DoF.) ${\varvec{c}}\in {R}^{DoF};$ ${\varvec{C}} \text{ is the set of all }{\varvec{c}}\text{; The }i^\text {th} \text{ element of } {\varvec{C}} \text{ is the movement label for the } i^\text{th} \text{ element of }{\varvec{S}}\text{.}$

*Normalize all elements of *$S$* to have L*^*1*^* norms of 1, generating *$\widehat{S}$* and *$\widehat{C}$*.* ${\Vert x\Vert }_{1}$ denotes the L^1^ norm for any vector $x$. $\text{Let }\overrightarrow{{{\varvec{s}}}_{{\varvec{i}}}} \text{ be the vector from } \left\{0\right\} \text{ to } {{\varvec{s}}}_{{\varvec{i}}} \in {\varvec{S}}\text{.}$; $\text{The corresponding element }\widehat{{{\varvec{s}}}_{{\varvec{i}}}} \in \widehat{{\varvec{S}}} \text{ is } \frac{{{\varvec{s}}}_{{\varvec{i}}}}{{\Vert \overrightarrow{{{\varvec{s}}}_{{\varvec{i}}}}\Vert }_{1}};$ The movement label for $\widehat{{{\varvec{s}}}_{{\varvec{i}}}}$ is $\widehat{{{\varvec{c}}}_{{\varvec{i}}}}=\boldsymbol{ }\frac{{{\varvec{c}}}_{{\varvec{i}}}}{{\Vert \overrightarrow{{{\varvec{s}}}_{{\varvec{i}}}}\Vert }_{1}};$ $\widehat{{{\varvec{c}}}_{{\varvec{i}}}} \in \widehat{{\varvec{C}}}$

*Expand *$\widehat{S}$* and *$\widehat{C}$*.* Activity on individual EMG channels appears on Cartesian basis vectors $\{{e}_{1},{e}_{2},\dots ,{e}_{n}\}$ in EMG feature space. $\stackrel{\sim }{{\varvec{S}}}$ is $\widehat{{\varvec{S}}}$ appended with $\{{e}_{1},{e}_{2},\dots ,{e}_{n}\}$ and {0}. $\stackrel{\sim }{{\varvec{C}}}$ is $\widehat{{\varvec{C}}}$ appended with $(n+1$) *DoF*-dimensional zero vectors: individual EMG channels and the origin are conservatively assigned movement labels indicating no movement.

*Partition space with Delaunay triangulation.* Delaunay triangulation tessellates EMG space into simplices emanating from the origin in a way that maximizes the minimum simplex angle using $\stackrel{\sim }{{\varvec{S}}}$ as a set of vertices. (Normalizing ${\varvec{S}}$ to a one-norm of one and including the origin forces simplices to emanate from zero.)

#### Online EMG evaluation (Fig. 2c, d)

For an incoming EMG signal, ${\varvec{p}}$, the controller must find the corresponding movement label ${{\varvec{c}}}_{{\varvec{p}}}$.

Scale $p$ to have an L^1^ norm of 1. $\widehat{{\varvec{p}}} =\frac{{\varvec{p}}}{{\Vert \overrightarrow{{\varvec{p}}}\Vert }_{1}}; \overrightarrow{{\varvec{p}}}\text{ being the vector from }\left\{0\right\}\text{ to }{\varvec{p}}\text{.}$

*Find the simplex containing *$\widehat{{\varvec{p}}}$

Simplices are parsed until the simplex containing $\widehat{{\varvec{p}}}$ is found.

Let ***T*** be the set of (*n* + 1) points bounding the simplex being parsed. ***T*** includes *n* points of $\stackrel{\sim }{{\varvec{S}}}$ and the origin. Let ${\varvec{U}}$ be the elements of $\stackrel{\sim }{{\varvec{C}}}$ which correspond to ${\varvec{T}}$; ***T*** and ${\varvec{U}}$ depend on the simplex.

$\widehat{{\varvec{p}}}$ can be described as a weighted sum of steady-state EMG activity from training data by:

$$\widehat{{\varvec{p}}}={k}_{1}{{\varvec{t}}}_{1}+\dots +{k}_{n+1}{{\varvec{t}}}_{n+1}; {{\varvec{t}}}_{i} \text{ being the }i^\text{th} \text{ point in } {\varvec{T}}; {k}_{1 },\dots , {k}_{n+1}\text{ scalars}$$

Solving this linear system yields ${k}_{1 },\dots , {k}_{n+1}$

If $\underset{i}{\text{min }}{k}_{i}\ge 0$ and $\sum_{1}^{{\varvec{n}}+1}{k}_{i}=1$, the correct partition has been identified.

*Interpolate to find *${{\varvec{c}}}_{\widehat{{\varvec{p}}}}$*.*

Once the correct partition has been found, linear interpolation determines ${{\varvec{c}}}_{\widehat{{\varvec{p}}}}$**:**

$${{\varvec{c}}}_{\widehat{{\varvec{p}}}}= {k}_{1}{{\varvec{u}}}_{1}+\dots +{k}_{n+1}{{\varvec{u}}}_{n+1}; {{\varvec{u}}}_{i} \text{ being the } i^\text{th} \text{ element of } {\varvec{U}};{k}_{1 },\dots , {k}_{n+1 } \text{ scalars}$$

*Scale *${c}_{\widehat{p}}$* by *$\Vert \overrightarrow{p}\Vert$* to find *${c}_{p}$*.*

$$c_{p} = \left( {\left\| {\vec{p}} \right\|_{1} } \right)*\left( {k_{1} u_{1} + \cdots + k_{n + 1} u_{n + 1} } \right)$$

$${{\varvec{u}}}_{i}\mathrm{ being the }i\mathrm{th element of }{\varvec{U}};{k}_{1 },\dots , {k}_{n+1 }\mathrm{scalars}$$

*Post-interpolation.* The output ${{\varvec{c}}}_{{\varvec{p}}}$ is an estimate of user effort in each DoF, with ‘a medium level of exertion’ corresponding to the value ‘1’. Two further steps convert user effort to hand velocity.

First, every actuated DoF is scaled in a physiologically-inspired mapping (Fig. 2d) to set nominal hand velocity [38]. The mapping is a smooth, piecewise curve consisting of four regions: very low, low, medium, and high effort. The regional mappings exhibit exponential, linear, square-root, and constant relationships, respectively. This effort-velocity mapping sets user activity below 50% of ‘a medium level of exertion’ to cap out at only 25% of maximum speed and penalizes effort exceeding ‘medium’. The mapping is meant to allow precise movements at lower EMG levels as well as quick movements at higher EMG amplitudes [39].

Second, prior to controller evaluation, the gains and thresholds for each degree of freedom are calibrated to user preference. This allows a “medium level of exertion” to correspond to any desired speed.

### Controller evaluation

Prosthetic hand controllers were evaluated by having subjects complete a target-matching task in the same VR environment used for controller training. Given the long training period needed to gather all training datasets and corresponding questions of user fatigue, as well as existing evidence of ciEMG controller stability, controllers were not evaluated on the same day as they were trained.

#### Target matching process

The target-matching task includes 80 targets in 5 batches of 16, with user-defined rest periods between batches. This number balances user fatigue against a thorough target selection [22].

To match a target posture, subjects use an EMG controller to move each DoF of a virtual hand to within 15% of the Range-of-Motion (RoM) of the target posture and then remain within that window for a continuous second. In our experience, subjects encounter difficulty in visualizing posture errors when tolerances are below 15% RoM (see Kinematic 4D videos—Additional file 2, Additional file 3). Subjects are given a 30 s time limit to match a target.

### Targets

Target postures span transitions in joint-movement space in a quasi-random manner:

First, generic movement directions are listed and shuffled. In 4-DoF, there are 80 generic movement directions (4-DoF, each of which can be − 1, 0 or 1, with ‘rest’ excluded). In the 3-Dof case, there are 26 generic movement directions. These were repeated thrice and, after shuffling, padded with two extra targets, excluded from analysis, to maintain the 80-target test length.

Target postures begin with a neutral hand posture, and subsequent postures are set by randomly changing each DoF based on the list of generic movement directions. No targets are within matching range of a range-of-motion limit. For example: if all DoF are limited between − 1 and 1, the matching window is 15% RoM, and the current generic movement direction is [1, − 1, 0] (a combined supination and wrist extension), the generated target will be at [rand(0.6,0.7), rand(− 0.6, − 0.7), 0]. If a target cannot be placed for the current generic movement direction, the next one is attempted. If none can be placed, the previous target is removed, the list of generic movement directions is circularly shifted, and the process proceeds from one target back.

This generates a set of targets that includes simultaneous movements in variable ratios and covers all generic movement directions without returning to a neutral posture. Target postures cover transitions requiring three or four combined movements despite training data only including single and paired movements.

#### Analysis

Target matching metrics are described in prior work [40]. Briefly, Match Percentage is the percentage of targets matched within the time limit. Time-To-Target is the time it takes a subject to match a target. Path Efficiency compares the travel path taken by the subject with the minimum straight-line travel path to the target posture. A 50% Path Efficiency, for example, indicates that the virtual hand traveled twice as far as strictly necessary to reach the target. Time-To-Target and Path Efficiency were only calculated for targets which were matched within the time limit. Path Efficiency excludes movement within the target matching window preceding a match (overshoots are accounted for), and Time-To-Target excludes the 1-s dwell time.

Match Percentages were compared using Fischer’s exact test. Time-To-Target and Path Efficiency were compared using two-way ANOVAs followed by paired t-tests. Bonferonni corrections were applied when comparing more than two data sets. Pearson’s Coefficients were used for determining the significance of trends over time. Significance was set at the p < 0.05 level. All metrics are written as mean ± standard error and drawn as mean with 95% CI.

#### Kinematic recordings

To provide subject-specific reference points, 3-DoF and 4-DoF target matching tasks were also repeated with the subjects’ intact limb. Electrogoniometer (Biometrics Ltd., Ladysmith, VA) recordings, with the fourth DoF controlled by thumb flexion/extension, were used.

In analyzing Path Efficiency with kinematic recordings, small-magnitude movements were adjusted to remove the effect of sensor noise. Pronation-supination recordings had a high resolution, and changes in sensor values were thresholded by assuming noise to be Gaussian around 0: a Normal curve was fitted to a 30-point histogram of frame-to-frame differences in sensor recordings, and the histogram window was adjusted until a minimal Root Mean Square Error was found for the fit. The threshold was set at three times the standard deviation of this fitted Normal curve, excluding movements below (0.12–0.34% RoM per 50-ms frame). Qualitatively, this approach excludes Gaussian low-magnitude movements. Wrist, finger, and thumb flexion–extension sensors had a lower resolution (around 0.17% RoM), and jitter between discretization levels was removed.

## Results

### Controller usability

During controller evaluation, subjects were given the option to declare a controller ‘unusable’ at any point in its evaluation. This would usually be decided while gains and thresholds were being tuned prior to controller evaluation, but sometimes occurred within the first few 16-target blocks. If a subject indicated that a controller was ‘unusable’, its evaluation would be de-prioritized for the day to minimize the effects of fatigue on other controllers being evaluated. Controllers which provided unreliable control over single-DoF movements were typically declared ‘unusable’.

Initially, both subjects found the 3-DoF (3D) controller and two of four 4-DoF controllers usable—one with the thumb controlled by a thumb movement (TH) and one with the thumb controlled by a radial-ulnar movement of the wrist (RU). (Table 2).

### 3-DoF performance over time

**Fig. 3.**
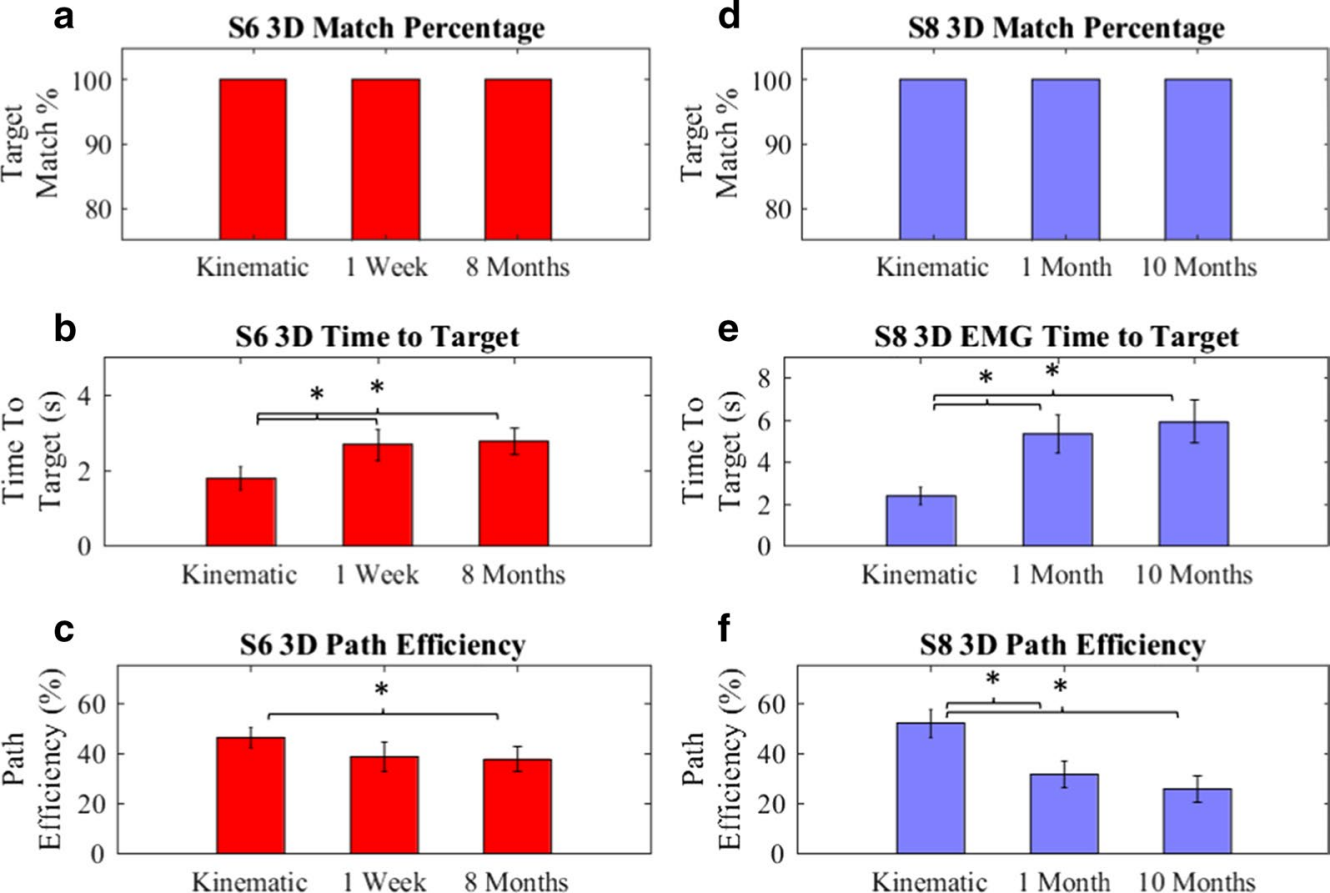
3-DoF performance over an 8–10 month period. S6 (Red, **a**–**c**) and S8 (Blue, **d**–**f**) performances in 3-DoF over an 8–10-month period are shown. (**a**, **d**) All targets were matched (**b**, **c**, **e**, **f**) EMG controllers were different (ANOVA, p < 0.05) from kinematic recordings, and showed no significant changes over 8–10 months. Values are presented as mean with 95% CI

3-DoF controllers were evaluated for both subjects shortly after training and again after a period of 8–10 months (Fig. 3). Neither EMG controller showed significant (p < 0.05) changes after this period.

*Subject S6*, with his intact limb, matched all targets averaging 1.80 ± 0.17 s/target and 46 ± 2% Path Efficiency. In initial tests, the EMG controller matched all targets averaging 2.69 ± 0.21 s/target and 39 ± 3% Path Efficiency.

*Subject S8*, with his intact limb matched all targets averaging 2.41 ± 0.21 s/target at a 52 ± 3% Path Efficiency. In initial tests, the EMG controller matched all targets averaging 5.36 ± 0.46 s/target at a 32 ± 3% Path Efficiency.

Anecdotally, S8′s movements appear to be less ballistic and more carefully planned than S6′s, potentially leading to slower match times (see S6 3-DoF video—Additional File 4; S8 3-DoF video, for data shown in Fig. 5,– Additional file 5).

### 4-DoF performance over time

**Fig. 4.**
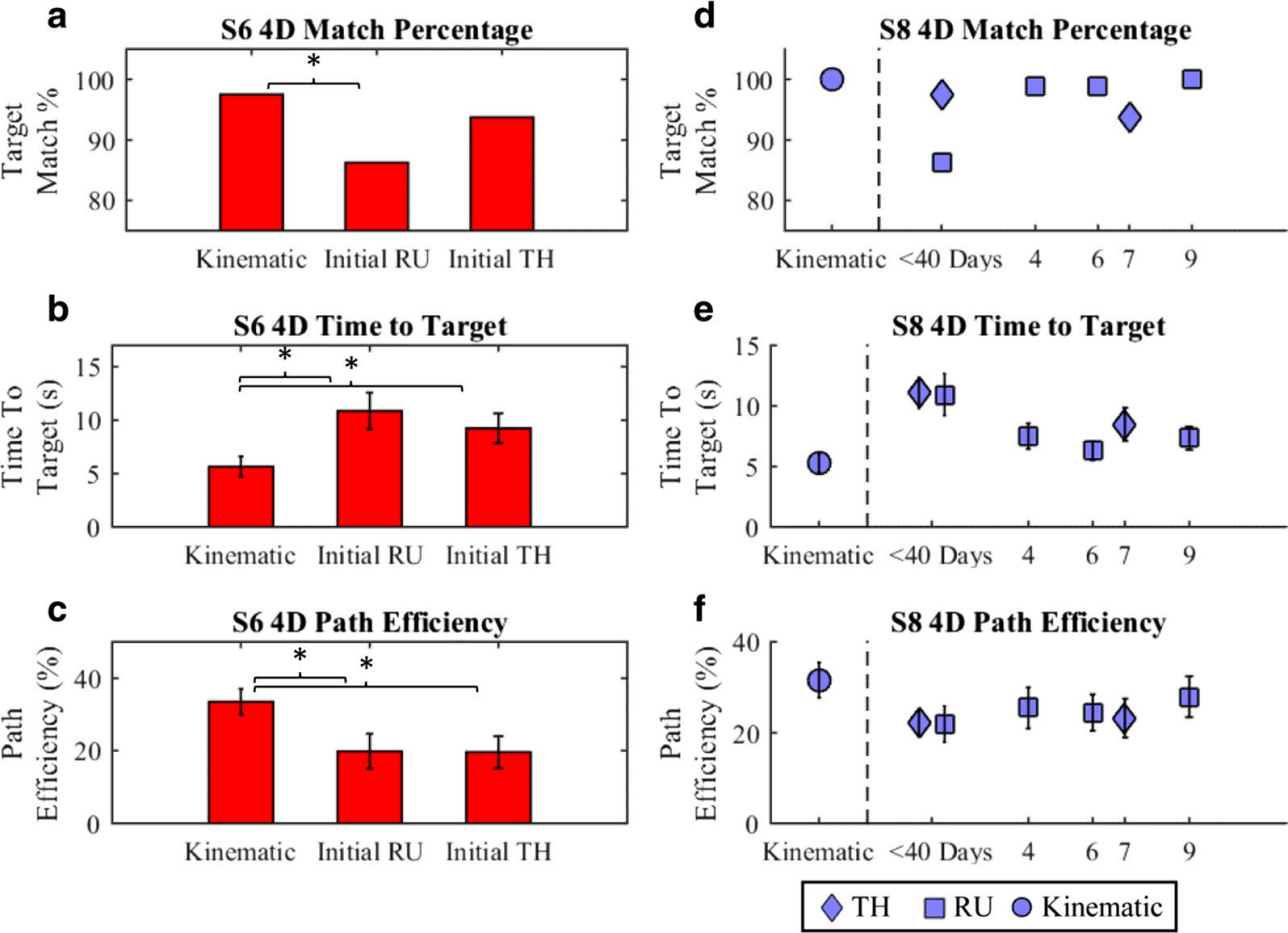
4-DoF performance over nine months. **a**–**c** Subject S6 4-DoF performance was unstable over time and differed from kinematic performance (paired t-test). **d**–**f** Subject S8 performance in 4-DoF over a 9-month period is shown. S8 RU showed an improving trend in Time-To-Target (Pearson, p < 0.05). S8 TH performance after 7 months showed significant improvement in Time-To-Target (paired t-test, p < 0.05). Comparisons to the S8 kinematic set were not explicitly drawn. All values are presented as mean with 95% CI

4-DoF controllers were evaluated for both subjects over a period of up to 9 months (Fig. 4). This evaluation shows trends that may indicate improvement over time without either retraining or at-home use.

*Subject S8* matched all targets with his intact limb averaging 5.28 ± 0.39 s/target at a 30 ± 2% Path Efficiency.

With one radial-ulnar 4-DoF EMG controller, S8 initially matched 86% of targets, averaging 10.94 ± 0.88 s/target at a 22 ± 2% Path Efficiency, then significantly improved in Time-to-Target and Match Percentage over the following 9 months (Pearson, p < 0.05). With an earlier 4-DoF RU controller (see 3.4), S8 matched 100% of targets, averaging 8.62 ± 0.64 s/target at a 34 ± 2% Path Efficiency.

With one thumb ab/adduction 4-DoF EMG controller, S8 initially matched 98% of targets, averaging 11.07 ± 0.65 s/target at a 22 ± 1% Path Efficiency; after 7 months S8 matched 94% of targets with this controller, averaging 8.48 ± 0.70 s/target at a 23 ± 2% Path Efficiency, significantly improving in Time-To-Target (paired t-test, p < 0.05). Although these S8 TH controllers were later found to be missing 2/32 training movements and the initial TH controller evaluation used a slightly different target set consisting solely of simultaneous movements in all 4-DoF, these changes did not affect performance as S8, with an earlier TH controller, matched 88% of targets averaging 12.42 ± 0.89 s/target at a 24 ± 2% Path Efficiency (see 3.4).

*Subject S6* performance was also evaluated in a 4-DoF case, despite the subject having fewer than 2xDoF EMG channels. With his intact limb, S6 matched 98% of targets averaging 5.66 ± 0.48 s/target at a 31 ± 2% Path Efficiency.

In initial tests with a 4-DoF thumb-flexion EMG controller, S6 matched 94% of targets, averaging 9.24 ± 0.71 s/target at a 20 ± 2% Path Efficiency. The controller was unstable upon re-evaluation after seven months.

While subject S6 did find the radial-ulnar 4-DoF EMG controller initially usable, matching 86% of targets and averaging 10.88 ± 0.88 s/target at a 20 ± 2% Path Efficiency, the controller was later found to be incorrectly trained and missing all paired movements involving radial-ulnar deviation (12/32 training sets). Although this controller was not usable on re-evaluation after seven months, no general conclusions about controller stability can be drawn from the RU evaluations.

### Reduced training dataset performance

**Fig. 5:**
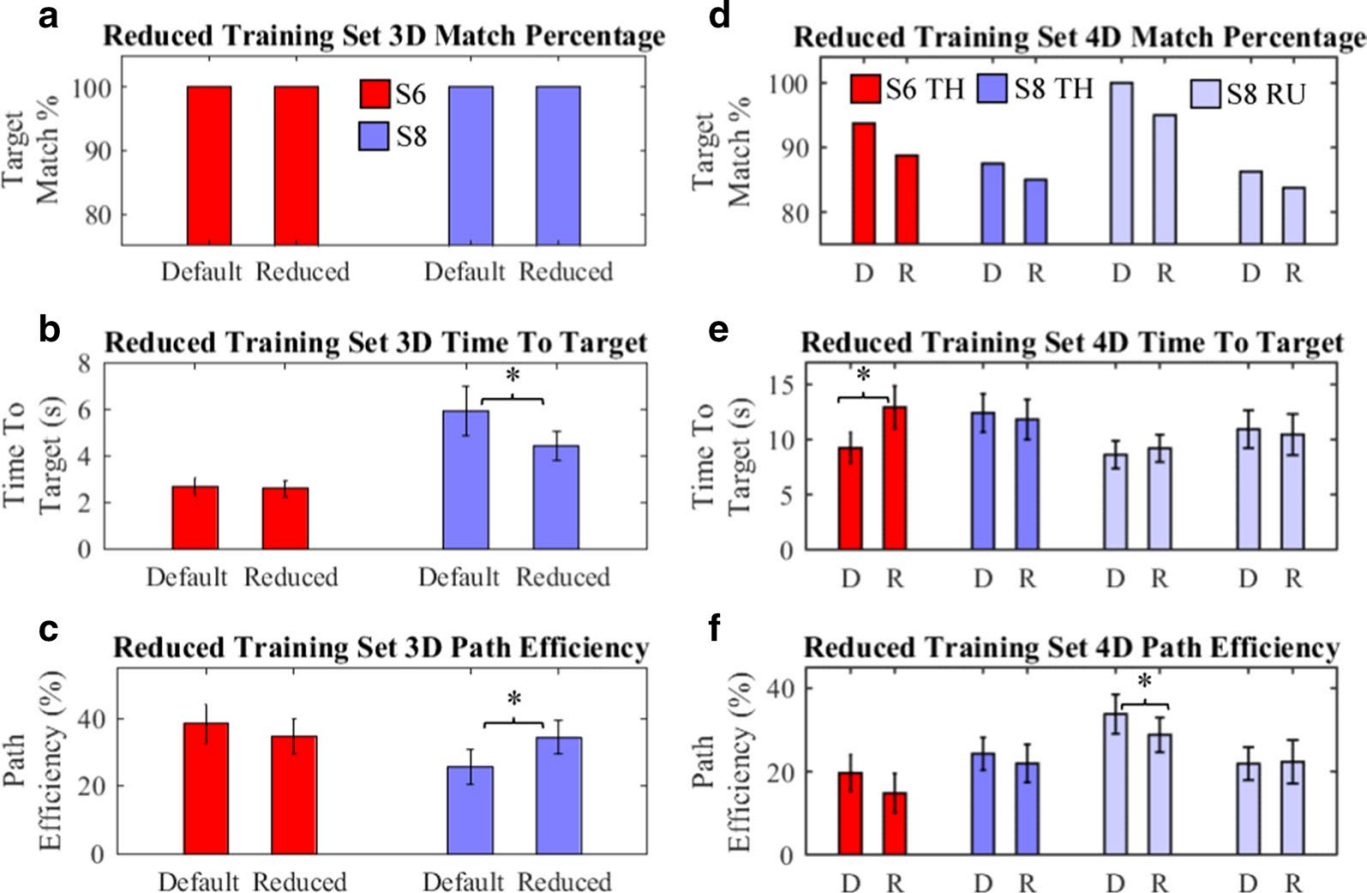
Reduced training dataset performance. S6 (Red) and S8 (Blue) performance with a default ‘D’ 3-of-5 repetition controller and reduced ‘R’ 1-of-5 repetition controller are shown. The S8 RU sets differ on whether the default case was run first (left) or the reduced case was run first (right), using different controllers evaluated 1 month post training. **a**, **d** No differences in Match Percentage were found (Fisher). **b**, **c** S8 3-DoF Time-to-Target and Path Efficiency improved with reduced repetitions (ANOVA, p < 0.05). **e** S6 Time-To-Target increased with the reduced set (paired t-test, p < 0.05). **f** Only one of two S8 RU sets indicated a significant (paired t-test) drop in Path Efficiency with a reduced training set. All values are presented as mean with 95% CI

One goal in prosthetics research is to reduce controller training time. The controller presented in this study builds a piecewise-linear model of user activity and needs an accurate, rather than large, training set. We hypothesized that controller performance with a reduced, single-best-repetition, training set would be comparable to performance with the default, multiple-repetition, training set. Default and reduced sets were compared to evaluate the controller’s performance with a minimal data set (Fig. 5). Default sets were run first. Order effect was examined in the 4-DoF S8 RU case, which was evaluated twice with different controllers 1 month post training, alternating whether the default or reduced case was run first.

*Default:* The default case (used in Figs. 3, 4) averages three of five movement repetitions to set the muscle pattern corresponding to a movement. This was done after accounting for possible user errors by removing two of five training repetitions with steady state patterns furthest from the mean movement pattern.

*Reduced:* The reduced case uses only one of the three ‘default’ repetitions—the repetition whose pattern is closest to the movement mean—to set the muscle pattern corresponding to a movement. Assuming that the distribution of EMG activity reduces with practice, shown in past studies [41, 42], this case represents the performance of a skilled user.

*In 3-DoF, subject S6′s* performance did not change with a reduced repetition set; subject S8′s Time-To-Target and Path Efficiency improved (paired t-test, p < 0.05; Video in Additional File 6).

*In 4-DoF, subject S8′s* performance did not change significantly with a reduced repetition training set, excepting a drop in Path Efficiency, but not other metrics, in the Default-Controller-First RU case. However, general conclusions on the interplay between training volume and Path Efficiency cannot be drawn as this was not observed in the second S8 4-DoF RU case. Missed S8 4-DoF targets were primarily composed of simultaneous 3-DoF and 4-DoF movements. Fatigue from successive evaluations does not appear to play a major role: in no case did the controller that was evaluated second have more than 20% of missed targets appear in the last of five batches.

*In 4-DoF, subject S6′s* Time-To-Target increased (paired t-test, p < 0.05) with a thumb-based 4-DoF controller; the reduced repetition radial-ulnar-based 4-DoF controller was unusable.

Results imply that the controller proposed in this study can function without performance loss, and possibly even with performance gains, in minimal-data conditions provided sufficient EMG channels. Under minimal-data conditions, training data collection times for the 32 recorded movements in a 4-DoF controller could be reduced to just under three minutes at five seconds per target.

## Discussion

We used linear interpolation, inspired by the synergy framework, with ciEMG electrodes to evaluate a novel simultaneous, intuitive, continuous, and proportional 4-DoF controller in VR.

When given at least two EMG channels per degree of freedom, the controller provides stable, possibly improving 3-DoF and 4-DoF control without retraining for up to 10 months. Past work indicates that strong VR controller performance carries over to functional benefit [43] and that continuous controllers can provide additional functional gain [21] relative to classifiers. Consequently, the presented controller may provide an avenue for improving the benefit that patients receive from modern prostheses. Additionally the controller demonstrated stable, possibly improving, Time-To-Target and Match Percentage metrics when trained on a one-movement-repetition training set provided sufficient EMG channels. While this is presently hypothesized to be of value only to skilled users, further examination is warranted.

### High-DoF control

This study demonstrated that linear interpolation, inspired by the synergy framework and using ciEMG electrode recordings, can provide 4-DoF simultaneous, continuous, intuitive, and proportional control in two subjects. The presented controller expands demonstrated continuous control beyond previously reported 2-DoF [12, 44] and 3-DoF [10, 30] cases. Furthermore, the controller examines a method of proportionality [29, 45] which only makes use of relevant EMG signals, although it does not quantify its effectiveness. As ciEMG is more localized than surface EMG [46] (sEMG), this approach may be less fatiguing than the traditional proportionality implementation of scaling velocity by the average global signal (e.g. Simon 2011 [19]).

While the controller takes roughly ten seconds to match a target in 4-DoF, these times should be placed in the context of the subject’s kinematic performance of roughly five seconds per target. First, this long kinematic match time implies that there is likely a heavy visualization component. Second, there appears to be a large practice component, as the best-performing S8 RU controller had a time-to-target of 6.3 ± 0.4 s/target (video in Additional File 7), compared to a kinematic time-to-target of 5.3 ± 0.4 s/target (video in Additional File 3). It is unclear how the 4-DoF controller would perform in activities of daily living without further investigation. Additionally, examining the target-matching videos leads to an interesting observation: “steady-state” activity is rare, and users start and stop frequently. The presented controller is built around the assumption of steady state EMG. We hypothesize that circumventing this assumption—such as by predicting steady-state EMG from transient EMG prior to interpolation—could improve performance.

### Stability and improvement

Few studies have engaged chronically implanted EMG to evaluate controller stability without retraining. In particular, Catalan [9] demonstrated stable control over three months with a 3-DoF classifier, Dewald [10] demonstrated stable control over three months with a 3-DoF regression, and Vu [47] demonstrated stable classification in 1–2 DoF over a 300-day period. This study demonstrates a controller capable of up to ten months of stable 3-DoF and up to nine months of stable, possibly improving, continuous 4-DoF control without retraining or at-home use through ciEMG recordings. This contrasts sharply with surface electrode controllers which need to be frequently retrained to maintain performance.

It is interesting to examine the changes in Subject S8′s RU 4D Time-to-Target over nine months. It appears that S8 becomes more consistent in matching targets despite months between controller evaluations (Table 3). This allows us to speculate on how the user’s default prosthesis controller impacts their use of the experimental controller. In particular, if the user’s controller had a detrimental impact, we would expect early batches to perform worse than later batches as the user becomes more acclimated to the experimental controller. Such acclimation is somewhat observed in the Kinematic case. RU cases, however, do not demonstrate consistent trends.

**Table 3:** Mean time-to-target for S8 4D RU controllers per test batch

Time-to-Target Average (s)						
Months Post-training	Batch number					Std. Dev.
	1	2	3	4	5	
1 Month*	6.6	11.6	8.4	9.5	12.5	2.4
1 Month	8.2	10.5	15.1	13.1	13.8	2.8
4 Months	7.0	7.7	8.0	9.6	10.3	1.4
6 Months	7.0	8.4	6.6	8.2	6.6	0.9
9 Months	7.9	6.8	10.1	8.5	8.7	1.2
Kinematic	7.3	6.5	5.7	5.7	6.1	0.6

Values in the central 6 × 5 grid are mean Time-To-Target, with missed targets excluded. The rightmost column shows the standard deviation across batches. Evaluations 4/6/9 months post-training appear to have more consistent rates between batches than both 1-month post-training evaluations

*From Sect. 3.4

### Controller behavior and low training time

A major advantage of the proposed controller is its piecewise-linear model of user EMG. This allows structured and predictable outputs, contrasting with ‘black-box’ approaches, and eliminates a reliance on training volume. As a result, neither Match Percentage nor Time-To-Target deteriorated when trained on one ‘best case’ repetition of each movement when provided with a sufficient number of EMG channels.

Low training times are frequently included in literature as a desired characteristic of prosthetic hand controllers [14, 21, 48]. The controller proposed in this study can potentially be trained in as little as three minutes, opening several directions for further research. First, rapid training times enable higher DoF controller development as even comprehensive high-DoF training data sets can be collected in just a few minutes. Second, small training times enable frequent, even daily, recalibrations and training set collections. In addition to simplifying training data collection in a take-home environment, this makes more complex studies on learning, EMG stability, and adaptive controllers, possible.

Although in practice such a controller will initially rely on a training set with multiple movement repetitions, the ‘best’ data tested here represents a reasonable prediction of the performance of a skilled user, as EMG activity becomes more consistent with use. While there is little recent research on EMG consistency, there is a demonstrated decrease in the coefficient of variability over a four-hundred repetition task [49] and an observed elimination of unwanted neural activity with practice [50]. With time, therefore, retraining this controller will only require a single repetition of each movement and take the user little time or effort. Determining how much practice, if any, a user requires to provide EMG signals sufficiently consistent for controller use warrants further investigation.

### Relationship between number of EMG channels and controllable DoF

Beyond the primary study conclusions, our data also suggests that the number of intuitively controllable DoF is not simply a function of the number of EMG channels. In (Table 2), only two of four evaluated 4-DoF controllers were deemed usable by either subject. The other two failed despite having a sufficient number of independent EMG channels. Additionally, a Monte-Carlo method was used to determine whether all movements were fundamentally reachable by subjects. 100,000 random velocity vectors were generated, and a corresponding EMG signal that would provide the requested velocity vector was found for every controller in Table 2. Hypothetically, every subject controller—except the under-trained S6 RU controller—was capable of providing a velocity vector in an arbitrary direction**.** The number of intuitively controllable DoF therefore appears to depend on the number, relevancy, and discernibility of user signals. In this regard, approaches such as TMR [52] or RPNI [45] may be beneficial. For example, recording signals from a thumb muscle would likely improve intuitive control of the thumb.

### Additional controller characteristics to be explored

Two aspects of the developed control algorithm provide additional, useful, customization options. First, the presented controller only implements a simple, unweighted interpolation where EMG patterns from known movements are combined in a linear manner to determine user’s intended movement. Other algorithms can be tuned to weight single-class movements preferentially or to implement features such as stiffness [52] or ‘positional wells’ [53].

Second, the effort–velocity curve converts intended movement effort into hand speed. We implemented this in a physiologically inspired relation, but any mapping can be used. Useful variants could emphasize fine movement control, stall hand speed when observing a stiffening co-contraction, or optimize hand acceleration.

As these customization steps are isolated from the controller’s ciEMG and homogeneity premise, benefits such as chronic stability or low-training-data requirements should carry over while evaluating new ways of tailoring controllers to tasks or users.

### Controller drawbacks

The controller has two primary drawbacks. First, the algorithm is not presently optimized and requires substantial computational resources during operation. These requirements increase combinatorially with more EMG channels and DoF. The calculation time is roughly 20 ms for a 4-DoF case on a modern (Intel i5-8250) processor. This calculation time is almost entirely devoted to a sequential search through simplices, whose number grows from 10,000 in a 4-DoF/8-EMG case to 200,000 in a 5-DoF/10-EMG case. Algorithmic optimization is necessary prior to deployment on embedded systems in 4 + -DoF or 10 + -EMG-Channel cases.

Second, the algorithm, as implemented, is likely only valid for use with implanted EMG. Linear interpolation assumes that ‘rest’ occurs when all EMG signals are near zero. Non-zero recordings during rest, which may be more prevalent with sEMG, would break this assumption. At present, we have not evaluated the controller’s performance with sEMG but expect that noise-floor-reduction techniques will be necessary to extend results to sEMG interfaces.

### Study limitations

This study is a case series and its greatest limitation is the small number of subjects, although all possible subjects with the appropriate implanted hardware were involved in the study. This number of subjects parallels past studies with implanted control and sensory restoration systems [2, 9, 10, 54, 55]. While the number of subjects is low, the volume of amputee subject data, covering 25 80-target evaluations, thoroughly examines subject performance and is not low compared to most prosthetics controls studies.

## Conclusions

The presented controller builds a linear model of user activity based on a synergy framework, reducing training time and implementing a synergy-based method of proportionality that may be more compatible with ciEMG recordings.

When provided with a sufficient number of EMG channels, the controller allowed subjects to match most targets in 3-DoF and 4-DoF posture-matching VR tasks; the controller also demonstrated stable, possibly improving, performance over 7–10 months without retraining, despite limited in-lab use. The controller also demonstrated stable Time-To-Target and Match Percentage when trained on minimal training data sets. Overall, the presented controller is an important step towards stable, High-DoF prosthesis control with short training times.

## Supplementary Information

**Additional file 1:** **Figure S1**

**Additional file 2:** Video of Subject S6 performing 4D posture-matching task with intact limb.

**Additional file 3:** Video of Subject S8 performing 4D posture-matching task with intact limb.

**Additional file 4:** Video of Subject S6 performing 3D posture-matching task 1 week post-training.

**Additional file 5:** Video of Subject S8 performing 3D posture-matching task with minimal training set controller 1 month post-training.

**Additional file 6:** Video of Subject S8 performing 3D posture-matching task with default training set controller 1 month post-training.

**Additional file 7:** Video of Subject S8 performing 4D posture-matching task 6 months post-training.

## Data Availability

Data will be made available upon request.

## Abbreviations

APT Center: Advanced Platform Technology Center
ciEMG: Chronically implanted EMG
DARPA: Defense Advanced Research Projects Agency
DoF: Degree of freedom (positive and negative)
ECRB: Extensor carpi radialis brevis
ECRL: Extensor carpi radialis longus
ECU: Extensor carpi ulnaris
ED: Extensor digitorum
EMG: Electromyography
FCR: Flexor carpi radialis
FCU: Flexor carpi ulnaris
FDS: Flexor digitorum superficialis
FES center: Functional electrical simulation center
HAPTIX: A DARPA Project, “Hand Proprioception and Touch Interfaces”
IDE: Investigational Device Exemption (Food and Drug Administration)
IRB: Institutional Review Board
RoM: Range of motion (for a joint in the prosthetic hand visualization)
RPNI: Regenerative peripheral nerve interfaces
RU: Refers to 4-DoF subject controller engaging ‘Radial-Ulnar’ movements
sEMG: Surface EMG
S6: A subject in the study, with 6 functional electrodes
S8: A subject in the study, with 8 functional electrodes
TH: Refers to 4-DoF subject controller engaging ‘Thumb’ movements.
TMR: Targeted muscle reinnervation
VR: Virtual reality
