# Supplementary material for: Stable, simultaneous and proportional 4-DoF prosthetic hand control via synergy-inspired linear interpolation: a case series
Source: J Neuroeng Rehabil. 2021 Mar 18;18:50. doi: 10.1186/s12984-021-00833-3 (PMC7977328; doi:10.1186/s12984-021-00833-3)
Supplement: Supplementary file 1 — Additional file 1: Figure S1 [file 12984_2021_833_MOESM1_ESM.pdf]

Additional File:
S8 3-DoF EMG patterns

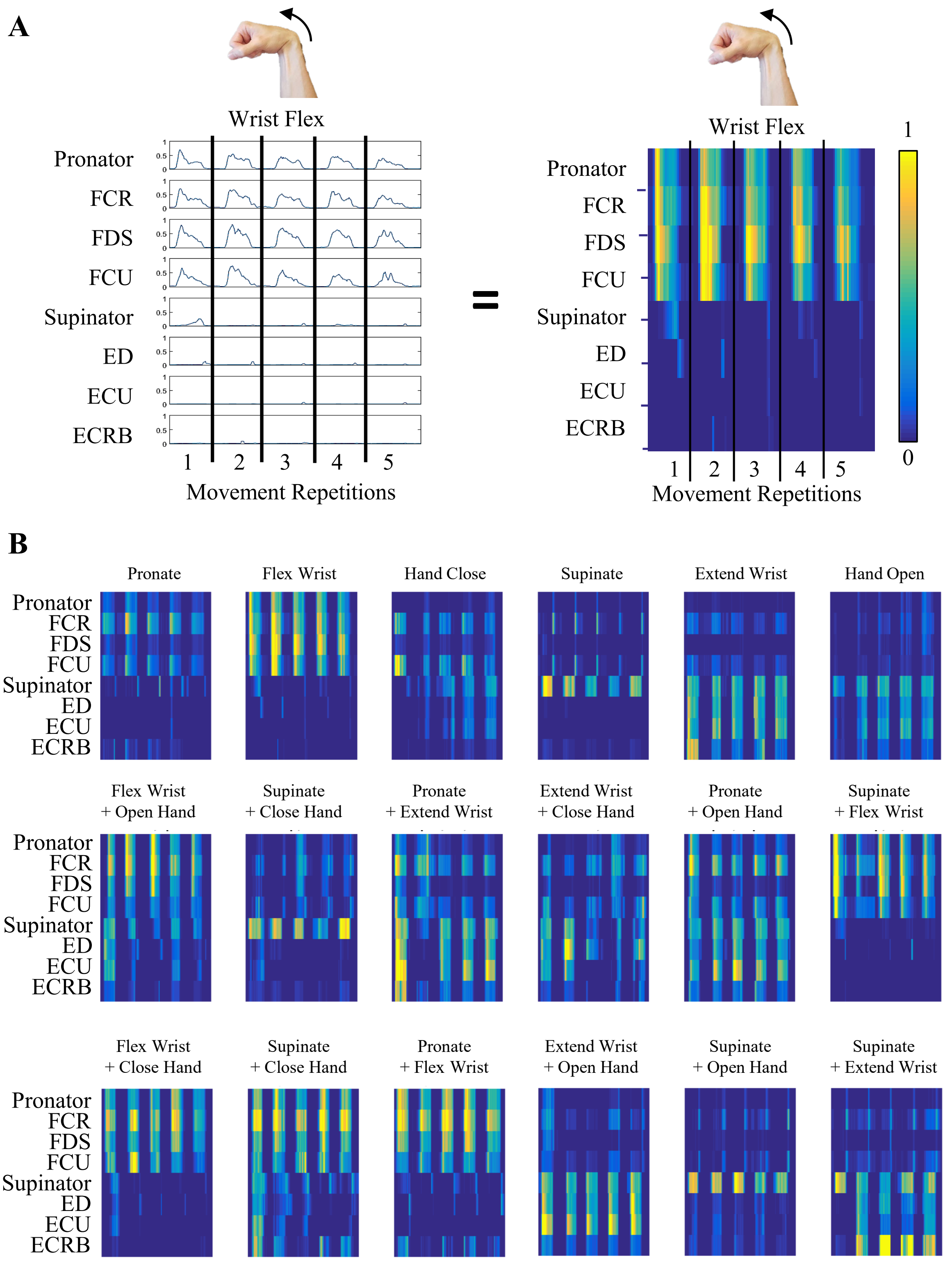

Supplementary Figure 1. S8 3-DoF EMG Patterns.
(A) S.F.1.A shows the EMG signal normalized per channel ('1' corresponding to the maximum EMG amplitude observed on a channel across the training data set) for five repetitions of the 'Wrist Flexion' movement from the S8 3-DoF training data set. This is also shown in an image form, with the color bar indicating normalized EMG magnitude. The color representation simplifies the intuitive comparison of 'EMG patterns' between movements (B) S.F.1.B depicts the image representation of each training movement (18 movements, 5 repetitions each).
